# Pyosalpinx causing acute appendicitis in a 32-year-old Cameroonian female: a case report

**DOI:** 10.1186/s13104-016-2175-3

**Published:** 2016-07-26

**Authors:** Valirie Ndip Agbor, Tsi Njim, Leopold Ndemnge Aminde

**Affiliations:** 1 Hope Clinic Bamenda, Bamenda, North west region Cameroon; 2 Nuffield Department of Medicine, University of Oxford, Oxfordshire, United Kingdom; 3 Health and Human Development (2HD) Research Group, Douala, Cameroon; 4 Clinical Research Education, Networking and Consultancy (CRENC), Douala, Littoral Cameroon; 5 School of Public Health, Faculty of Medicine and Biomedical Sciences, University of Queensland, Brisbane, Australia

**Keywords:** Pyosalpinx, Acute appendicitis, Cameroon

## Abstract

**Background:**

Pyosalpinx as a cause of acute appendicitis is very rare. We report the first case of a right pyosalpinx causing an extrinsic compression of the appendiceal lumen and consequently an acute appendicitis following an obstruction.

**Case presentation:**

A 32 year old female from the North west region of Cameroon, presented with an acute exacerbation of a mild chronic right iliac fossa pain over a 2 day duration. She underwent exploratory laparotomy and intraoperative findings were a bilateral pyosalpinx, with the right fallopian tube adhering to the caecum and the terminal ileum, and obstructing the lumen of an inflammed appendix. A classical appendectomy and a right salpingectomy were done. The post-operative period was uneventful and she returned after 2 weeks for a follow-up visit with no further complaints.

**Conclusion:**

We describe to the best of our knowledge, the first case of an acute appendicitis caused by an extrinsic obstruction of the appendiceal lumen by a pyosalpinx. The close proximity of the caecum to the right fallopian tube most likely accounted for this occurrence. Although a rare entity, physicians should always keep in mind very rare causes of an acute appendicitis to guide management. This case highlights the shortcomings of pelvic ultrasonography in the diagnosis this condition. A pelvic computed tomography scanning should therefore be sought in case of a doubtful pelvic ultrasonography result. Finally, there is an urgent need to improve the awareness on sexually transmitted infections in our setting.

## Background

Acute appendicitis is the commonest cause of acute abdominal pain, representing 27.5 % of all abdominal surgical emergencies, with an estimated life-time risk of 7–8 % [1, 2]. Acute appendicitis is most commonly treated through emergency appendectomy [2, 3]. The most common cause of acute appendicitis is obstruction of the appendiceal lumen secondary to fecoliths in adults and lymphoid hyperplasia in children [4].

Pyosalpinx is the collection of pus in the fallopian tubes, and sexually transmitted infections (STIs) such as *Chlamydia trachomatis* and *Neisseria gonorrhea,* as well as some enteric bacteriaceae have been implicated [5, 6]. Pyosalpinx is a severe sequelae of chronic pelvic inflammatory disease (PID) and occurs frequently in women between the ages of 20–40 years even though it can occur in older and younger women. It complicates 16.1 % of all cases of PID (annual incidence of PID in women of reproductive age in the United States is 200,000 cases) [6, 7]. The complications of pyosalpinx vary from secondary infertility, to ectopic pregnancies [8], and secondary peritonitis from a ruptured pyosalpinx [9]. Furthermore, Jackson and coworkers previously reported a case of acute appendicitis complicating a torsioned and gangrenous pyosalpinx most probably secondary to a contiguous infection [10].

We herein, describe to the best of our knowledge, the first case of an acute appendicitis caused by an extrinsic obstruction of the appendiceal lumen by a pyosalpinx.

## Case presentation

A 32 year old married African female from the North west region of Cameroon, G4P4004 (four previous pregnancies, four term deliveries, no abortion, no preterm deliveries, and four living children), with a history of mild recurrent right iliac fossa (RIF) pain presented at our emergency service with an acute exacerbation of the pain over a 2 day duration and an episode of post prandial vomiting. She reported anorexia, and a low grade fever. She had no past history of a sexually transmitted infection.

On examination, she was ill looking, tachycardic (pulse rate = 144 beats per minute) and febrile (maximum temperature = 38.4 °C). She had a RIF tenderness, and positive Blumberg’s (Rebound tenderness at the RIF), Rovsing’s (increased pain at the RIF on deep palpation of the left iliac fossa), Obturator (abdominal pain on flexion and internal rotation of the hip) and Psoas (abdominal pain on passive extension or active flexion of the thigh at the hip) signs.

A full blood count revealed a leucocytosis at 13,000 cells/mm^3^. A urinalysis and stool exam were normal and a pregnancy test was negative. The patient had a total Alvarado’s score of 10 and a diagnosis of an acute appendicitis was further advocated by an abdominopelvic ultrasound which showed; an indistinct mass in the RIF, an inflammed appendix and collection of fluid in the pouch of Douglas. A diagnosis of an acute appendicitis associated with an indistinct RIF fossa mass was proposed, and an exploratory laparotomy via a midline infra-umbilical incision was performed.

**Fig. 1:**
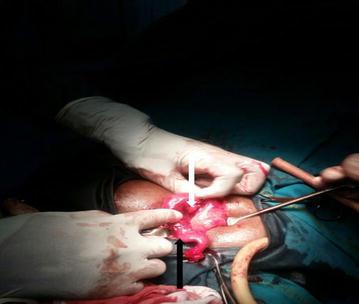
Intraoperative view of right pyosalpinx (*black arrow*) adhered to the terminal ileum and caecum obstructing the lumen of an inflammed appendix (*white arrow*)

**Fig. 2:**
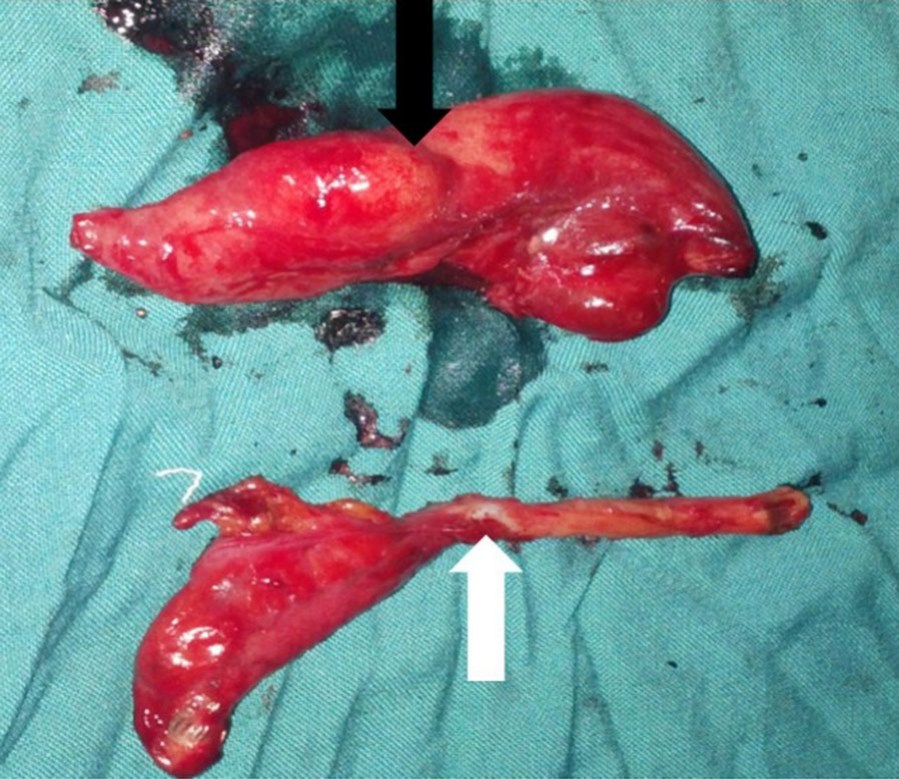
Resected pieces of a right fallopian tube (*black arrow*) and appendix (*white arrow*)

Intraoperatively, an inflammed, suppurative, and distorted right fallopian tube measuring 8 × 4 cm, attached to the caecum and the terminal ileum by means of pseudo-membranes was found compressing and occluding the lumen of an inflammed appendix (Figs. 1, 2). There was extensive damage of the right tube with complete obliteration of its lumen rendering saline irrigation and intraluminal adhersiolysis impossible. The left fallopian tube was also inflammed, measuring 4 × 4 cm and discharging pus. The ovaries and uterus were macroscopically normal. A right salpingectomy and classical appendectomy were done. The pus from the left fallopian tube was drained, and the tube irrigated with normal saline followed by abdominal lavage. A pus sample was collected but could not be analysed due to financial constraints.

The patient was therefore placed on intravenous (IV) antibiotics and the post-operative period was uneventful. The patient had positive Chlamydia Immunoglobulin M titres and a negative TPHA (Treponema Pallidum Haemagglutination Assay) test. She and her spouse were treated with doxycycline 200 mg once daily and metronidazole 500 mg 8 hourly upon discharge to complete a 14 day course of treatment. She returned for a follow up visit 2 weeks later with no further complaints.

## Discussion

Obstruction of the appendiceal lumen is the most common cause of acute appendicitis. Obstruction of the appendiceal lumen can be classified into; intraluminal (fecolith, parasites) [4, 11], intramural (lymphoid hyperplasia, enterobiasis and carcinoid tumours) [4, 11], and rarely, extramural (metastatic cervical cancer) [12]. Although fecoliths and lymphoid hyperplasia remain the most common causes of obstruction, unusual causes of obstruction also have a role to play in 1 % of cases with carcinoid tumours and *Enterobius vermicularis* occupying the first two ranks [11]. Pyosalpinx as a cause of appendicitis is very rare. A MEDLINE and Google scholar search from inception to date yielded just two similar cases of an acute appendicitis complicating a pyosalpix (Table 1). These were due to contiguous infection from a suppurating pyosalpinx. The proximity of the caecum to the right fallopian tube, most likely accounted for this occurrence.

**Table 1:** Comparison of our case with similar cases in terms of the proposed mechanism, pathology (per-operatively), treatment and outcome

Author	Mechanism	Pathology	Treatment	Postoperative outcome
Jackson [10]	Contiguous infection	Oedematous appendix Torsioned and Gangrenous right pyosalpinx measuring 12.5 cm by 5 cm	Appendicectomy Right salpingectomy Closure without drainage It was not mentioned if patient was placed on intravenous antibiotics	Uneventful
Halpenny [20]	Contiguous infection from a right pyosalpinx	Right fallopian tube was found discharging pus. State of the tube not mentioned Left tube was normal Inflammed and adherent appendix	Appendicectomy Right salpingectomy Abdominal toileting Closure without drainage Patient received intravenous antibiotics	Uneventful
Our case	Extrinsic compression of the appendix by a right pyosalpinx	Distorted right fallopian tube measuring 8 by 4 cm and discharging pus Inflammed left fallopian tube discharging pus Inflammed appendix	Right salpingectomy Appendicectomy Drainage and saline irrigation of the left fallopian tube Abdominal lavage intravenous antibiotics	Uneventful

To the best of our knowledge, this is first case of appendicitis secondary to a mechanical compression by a pyosalpinx.

STIs such as *Chlamydia trachomatis* and *Neisseria gonorrhea,* as well as some enteric bacteriaceae have been implicated as the most frequent causes of a pyosalpinx [5, 6]. However, pelvic surgeries and intra-abdominal infections are recognised culprits [6]. Furthermore, some germs could be introduced to the upper genital tract during iatrogenic procedures like abortions, childbirth and when placing an intra-uterine contraceptive device [6]. Nevertheless, there have been reports of pyo/hydrosalpinx in virginal adolescents with genitourinary malformations [13] and in prepubescent females with Hirschprung’s disease [14].

The signs and symptoms of pyosalpinx include pelvic pains, fever, and a palpable pelvic mass on bimanual examination. In some instances, it may present with features of paralytic ileus and bowel obstruction. When the right fallopian tube is affected, many of these signs mimic those of acute appendicitis, creating a diagnostic dilemma [7].

Generally, ultrasonography is the preferred technique in diagnosing pelvic masses. Computed tomography (CT) scanning is usually preserved for cases of inconclusive ultrasonography results, while magnetic resonance imaging (MRI) is used to characterise complex masses [15]. Studies have shown the superiority of CT scanning over ultrasonography in the diagnosis of acute appendicitis and also, the former readily provides an alternative diagnosis since it views the entire abdomen [16, 17]. CT scan machines are not readily available in a resources-limited setting like ours, it was therefore difficult to make a definitive preoperative diagnosis of an appendicitis complicating a pyosalpinx.

The gold standard for management of acute appendicitis generally involves open appendectomy, while laparoscopic appendectomy has specific indications [18]. On the other hand, a wide range of interventions have been described for the management of pyosalpinx; from intravenous antibiotics, to laparoscopic aspiration or laparoscopic salpingotomy with saline irrigation, and/or drainage, and salpingectomy [19]. Salpingectomy is usually reserved as a last resort in premenopausal women. We opted for a laparotomy in our patient to explore the RIF mass suggested in the ultrasound and more so, the financial situation of our patient didn’t permit access to a laparoscopic management which is not readily available in resource-limited settings. Due to the extensive affection of the right tube, we went for a right salpingectomy after intraoperative consent was obtained from her spouse.

There is an urgent need to improve awareness on STIs in our setting as the woman in our case had been experiencing a recurrent RIF pain for a number of years and never sought medical attention.

## Conclusion

We have described the first case of acute appendicitis secondary to extrinsic mechanical obstruction of the appendiceal lumen by a right pyosalpinx. Physicians should keep a high index of suspicion for the very rare causes of acute appendicitis, supported by in-depth clinical examination and investigation. Though our economically disadvantaged setting precluded optimum care, the place of an exploratory laparotomy in such circumstances cannot be completely underscored.

## Abbreviations

RIF: right iliac fossa
TPHA: Treponema Pallidum Haemagglutination Assay
CT: computed tomography
MRI: Magnetic Resonance Imaging
IV: intravenous
STIs: sexually transmitted infections
